# Strategies for effective dissemination of research to United States policymakers: a systematic review

**DOI:** 10.1186/s13012-020-01046-3

**Published:** 2020-10-15

**Authors:** Laura Ellen Ashcraft, Deirdre A. Quinn, Ross C. Brownson

**Affiliations:** 1 University of Pittsburgh School of Social Work, 2117 Cathedral of Learning, 4200 Fifth Avenue, Pittsburgh, PA 15260 USA; 2 Center for Health Equity Research and Promotion (CHERP), VA Pittsburgh Healthcare System, University Drive C, Building 30, Pittsburgh, PA 15240 USA; 3 Prevention Research Center, Brown School, Washington University in St. Louis, One Brookings Drive, Campus Box 1196, St. Louis, MO 63130 USA; 4 Department of Surgery, Division of Public Health Sciences, and Alvin J. Siteman Cancer Center, Washington University School of Medicine, 660 South Euclid Avenue, Saint Louis, MO 63110 USA

**Keywords:** Systematic review, Dissemination, Dissemination science, Social policy, Public policy

## Abstract

**Background:**

Research has the potential to influence US social policy; however, existing research in this area lacks a coherent message. The Model for Dissemination of Research provides a framework through which to synthesize lessons learned from research to date on the process of translating research to US policymakers.

**Methods:**

The peer-reviewed and grey literature was systematically reviewed to understand common strategies for disseminating social policy research to policymakers in the United States. We searched Academic Search Premier, PolicyFile, SocINDEX, Social Work Abstracts, and Web of Science from January 1980 through December 2019. Articles were independently reviewed and thematically analyzed by two investigators and organized using the Model for Dissemination of Research.

**Results:**

The search resulted in 5225 titles and abstracts for inclusion consideration. 303 full-text articles were reviewed with 27 meeting inclusion criteria. Common sources of research dissemination included government, academic researchers, the peer reviewed literature, and independent organizations. The most frequently disseminated research topics were health-related, and legislators and executive branch administrators were the most common target audience. Print materials and personal communication were the most common channels for disseminating research to policymakers. There was variation in dissemination channels by level of government (e.g., a more formal legislative process at the federal level compared with other levesl). Findings from this work suggest that dissemination is most effective when it starts early, galvanizes support, uses champions and brokers, considers contextual factors, is timely, relevant, and accessible, and knows the players and process.

**Conclusions:**

Effective dissemination of research to US policymakers exists; yet, rigorous quantitative evaluation is rare. A number of cross-cutting strategies appear to enhance the translation of research evidence into policy.

**Registration:**

Not registered.

Contributions to the literature

- This is one of the first systematic reviews to synthesize how social policy research evidence is disseminated to US policymakers.
- Print materials and personal communications were the most commonly used channels to disseminate social policy research to policymakers.
- Several cross-cutting strategies (e.g., start early, use evidence “champions,” make research products more timely, relevant, and accessible) were identified that are likely to lead to more effective translate of research evidence into the policy making process in the United States.

## Background

In recent years, social scientists have sought to understand how research may influence policy [1, 2]. Interest in this area of investigation has grown with the increased availability of funding for policy-specific research (e.g., dissemination and implementation research) [3]. However, because of variation in the content of public policy, this emerging area of scholarship lacks a coherent message that specifically addresses social policy in the United States (US). While other studies have examined the use of evidence in policymaking *globally* [4–7], the current review focuses on US social policy; for the purposes of this study, social policy includes policies which focus on antipoverty, economic security, health, education, and social services [8–10].

Significant international research exists on barriers and facilitators to the dissemination and use of research evidence by policymakers [4, 5]. Common themes include the importance of personal relationships, the timeliness of evidence, and resource availability [4, 5]. Previous work demonstrates the importance of understanding policymakers’ perceptions and how evidence is disseminated. The current review builds on this existing knowledge to examine *how* research evidence reaches policymakers and to understand what strategies are likely to be effective in overcoming identified barriers.

Theoretical frameworks offer a necessary foundation to identify and assess strategies for disseminating research to policymakers. The Model for Dissemination of Research integrates Diffusion of Innovations Theory and Social Marketing Theory with the Mathematical Theory of Communication [11, 12] and the Matrix of Persuasive Communication [13, 14] to address the translation gap between research and policy. The purpose of the Model for Dissemination of Research is to highlight the gaps between research and targets audiences (e.g., policymakers) and improve dissemination through the use of a theoretical foundation and review of the literature [15]. Diffusion of Innovations Theory describes the spread and adoption of novel interventions through an “s-curve,” ordered process, and characteristics of the message and audience [16]. Additional theoretical contributions for dissemination research come from Social Marketing Theory, which postulates commercial marketing strategies summarized by the four P’s (produce, price, place, and promotion) and the understanding that communication of the message alone will not change behavior [17].

**Fig. 1:**
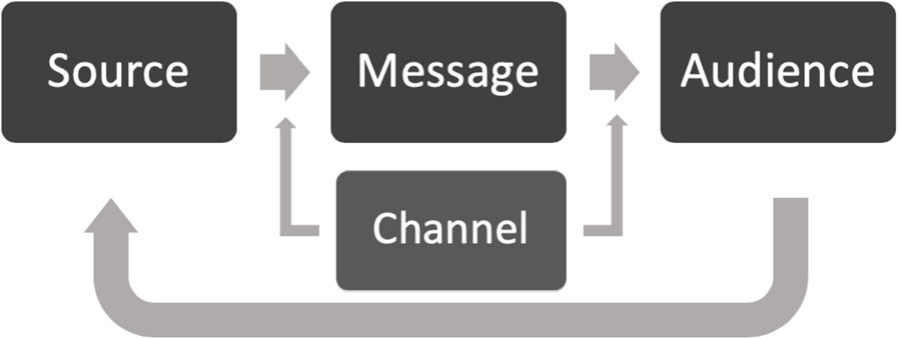
The Model for Dissemination of Research. The Model for Dissemination of Research integrates Diffusion of Innovations Theory, the Mathematical Theory of Communication, and Social Marketing Theory to develop a framework for conceptualizing how information moves from source to audience. Originally published by Brownson et al. in Journal of public health management and practice in 2018

The Model for Dissemination of Research includes the four key components described by Shannon and Weaver [11, 12] and later McGuire [13, 14] of the research translation process: the source, message, audience, and channel (Fig. 1). The *source* includes researchers who generate evidence. The *message* includes relevant information sent by the source on a policy topic. The *audience* includes those receiving the message via the channel [15]. The *channel* is how the message gets from the source to the audience [15].

While the Model for Dissemination of Research and its origins (i.e., the Mathematical Theory of Communication and Diffusion of Innovations Theory) appear linear in their presentation, Shannon and Weaver [11, 12] and Rogers [16] clearly acknowledge that the dissemination of information is not a linear process and is effected by the environment within which it occurs. This approach aligns with the system model or knowledge to action approach proposed by Best and Holmes [18]. The systems model accounts for influence of the environment on a process and accounts for the complexity of the system [18]. Therefore, while some theoretical depictions appear linear in their presentation; it is important to acknowledge the critical role of systems thinking.

To date, lessons learned from dissemination and implementation science about the ways in which research influences policy are scattered across diverse disciplines and bodies of literature. These disparate lessons highlight the critical need to integrate knowledge across disciplines. The current study aims to make sense of and distill these lessons by conducting a systematic review of scientific literature on the role of research in shaping social policy in the United States. The results of this systematic review are synthesized in a preliminary conceptual model (organized around the Model for Dissemination of Research) with the goal of improving dissemination strategies for the translation of scientific research to policymakers and guiding future research in this area.

This systematic review aims to synthesize existing evidence about how research has been used to influence social policy and is guided by the following research questions:

1. What are common strategies for using research to influence social policy in the United States?
2. What is the effectiveness of these strategies?

## Methods

We used the Preferred Reporting Items for Systematic Reviews and Meta-Analyses (PRISMA-P) model [19, 20] to examine and distill existing studies on strategies for using research evidence to influence social policy.

### Eligibility criteria

Studies were eligible for this review if they met the following inclusion criteria: (1) occurred in the United States; (2) reported in English; (3) systematically evaluated the impact of research on social policy (this typically excluded studies focusing on policymaker dissemination preferences); (4) discussed domestic social policy (as defined above); and (5) were published in the peer reviewed literature or the grey literature (e.g., think tank research briefs, foundation research publications).

We chose to focus our review on the United States to capture the strengths and challenges of its unique, multi-level policy and political environment. The de-centralized structure of government in the United States allows significant decision-making authority at the state and local levels, with wide variation in capacity and the availability of resources across the country [21]. For example, some states have full-time legislatures while other states have part-time legislatures. In total, these factors create a fitting and complex environment to examine the dissemination of research to policymakers. The influence of lobbying in the United States also differs from other western countries. In the United States, there is more likely to be a “winner-take-all” process where some advocates (often corporations and trade associations) have disproportionate influence [22]. In addition, the role of evidence differs in the US compared with other countries, where the US tends to take a narrower focus on intervention impact with less emphasis on system-level issues (e.g., implementation, cost) [23].

Studies were excluded if they were not in English or occurred outside of the United States. We also excluded non-research sources, such as editorials, opinion pieces, and narrative stories that contain descriptions of dissemination strategies without systematic evaluation. Further, studies were excluded if the results focused on practitioners (e.g., case managers, local health department workers) and/or if results for practitioners could not be parsed from results for policymakers.

To identify studies that systematically evaluated the impact of research on social policy, we reviewed the research questions and results of each study to determine whether or not they examined *how* research evidence reaches policymakers (as opposed to policymaker preferences for disseminated research). For example, we would not include a research study that only describes different types of policy briefs, without also evaluating how the briefs are used by policymakers to inform policy decisions. We used the Model for Dissemination of Research, as defined above, to see if and how the studies describe and test the channels of dissemination. We built on the Model of Dissemination by also considering passive forms of knowledge, such as peer-reviewed literature or research briefs, as potential sources of knowledge and not just as channels in and of themselves.

### Information sources

We took a three-pronged approach to develop a comprehensive understanding of existing knowledge in this area. First, we searched the peer reviewed literature using the following databases: Academic Search Premier, PolicyFile, SocINDEX, Social Work Abstracts, and Web of Science. We expanded the inquiry for evidence by searching the grey literature through PolicyFile, and included recommendations from experts in the field of dissemination of research evidence to policymakers resulting in 137 recommended publications.

### Search strategy

Our search strategy included the following terms: [research OR study OR studies OR knowledge] AND [policy OR policies OR law OR laws OR legislation] AND [use OR utilization OR utilisation] OR [disseminate OR dissemination OR disseminating] OR [implementation OR implementing OR implement] OR [translate OR translation OR translating]. Our search was limited to studies in the United States between 1980 and 2019. We selected this timeframe based on historical context: the 1950s through the 1970s saw the development of the modern welfare state, which was (relatively) complete by 1980. However, shifting political agendas in the 1980s saw the demand for evidence increase to provide support for social programs [24]; we hoped to capture this increase in evidence use in policy.

### Selection process

All titles and abstracts were screened by the principal investigator (LEA) with 20% reviewed at random by a co-investigator (DAQ) with total agreement post-training. Studies remaining after abstract screening moved to full text review. The full text of each study was considered for inclusion (LEA and DAQ) with conflicts resolved by consensus. The data abstraction form was developed by the principal investigator (LEA) based on previous research [25, 26] and with feedback from co-authors. Data were independently abstracted from each reference in duplicate with conflicts resolved by consensus (LEA and DAQ). We completed reliability checks on 20% of the final studies, selected at random, to ensure accurate data abstraction.

### Data synthesis

Abstracted data was qualitatively analyzed using thematic analysis (LEA and DAQ) and guided by the Model for Dissemination of Research. The goal of the preliminary conceptual model was to synthesize components of dissemination for studies that evaluate the dissemination of social policy to policymakers.

## Results

### Descriptive results

**Fig. 2:**
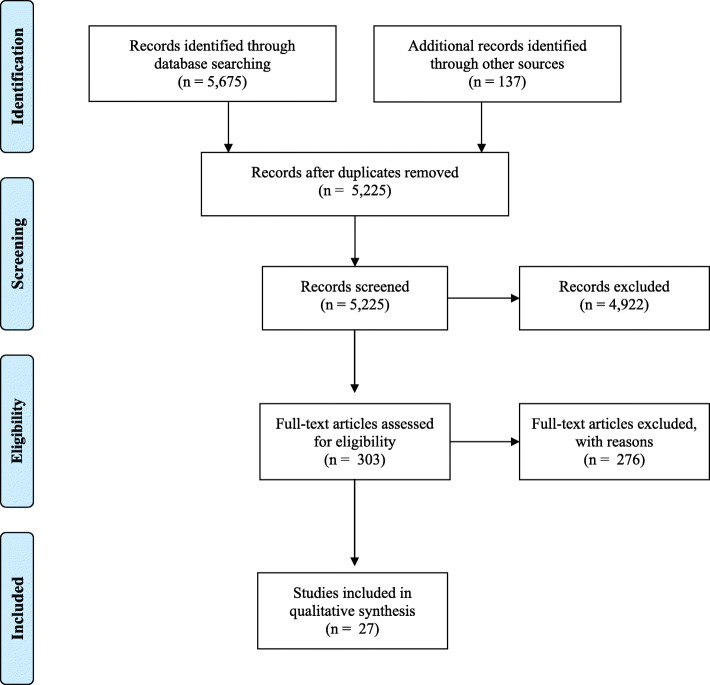
PRISMA flowchart. The preferred reporting items for systematic reviews and meta-analyses (PRISMA) flow diagram reports included and excluded articles in the systematic review

The search of the literature resulted in 5675 articles and 137 articles recommended by content experts for review with 5225 titles and abstracts screened after duplicates removed. Of those articles, 4922 were excluded due to not meeting inclusion criteria. Further, 303 full text articles were reviewed with 276 excluded as they did not meet inclusion criteria. Twenty-seven articles met inclusion criteria (see the Fig. 2 for the PRISMA flow diagram).

Included studies are listed in Table 1. The 27 included 6 studies using quantitative methods, 18 that employed qualitative methods, and 3 that used a mixed methods approach. The qualitative studies mostly employed interviews (*n* = 10), while others used case studies (*n* = 6) or focus groups (*n* = 3). Most studies examined state-level policy (*n* = 18) and nine studies examined federal-level policy, with some studies looking at multiple levels of government. Included studies focused on the executive and legislative branches with no studies examining the judicial branch.

**Table 1:** Included studies

Lead author, Year	Theory/framework	Method and sample size	Level and branch of government	Source (i.e., where/who is the information coming from)	Message (i.e., what information is being shared)	Channel (i.e., how is the information getting to the target audience)	Audience (i.e., who is the information going to)
Allen, 2015 [45]	The operationalization framework	Qualitative; 29 Interviews	Local/Municipal	Policymakers and advocates from three cities	Information about needle exchanges	Public education campaign about needle exchange; public pressure put on policymakers’	Policymakers (broadly defined)
Austin, 2017 [47]	The 'triggers-to-action' framework	Qualitative (case study)	State; Legislative	An academic-community-government partnership	Harm caused to consumers by dietary supplements for weight loss and muscle building	Peer-reviewed recommendations; Media outreach campaign, via websites, email listservs and social media; Fact sheet, talking points, summary of research, the original legal research article	State legislators
Brim, 1983 [46]	None	Qualitative (case study)	Other; Legislative	Foundation Reports Child Development	Current state and past trends for children and families; budget analysis; national survey; federal expenditures	Reports; books; collaboration between foundations and the public sector; grants to advocacy organizations; compilation of data	Senate Subcommittee on Children and Youth Hearings
Brownson, 2011 [38]	None	Quantitative; 291	State; Executive, Legislative	State-level policy makers	Information about mammography screening	Policy briefs (data/state; data/local; story/state; story/local)	State-level policymakers
Bumbarger, 2012 [48]	Interactive Systems Framework	Qualitative (case study)	State; Executive	Penn State Prevention Research Center program staff and researchers	Ongoing information about evidence-based practices and policies	One-page fact sheets, PowerPoint presentations, research briefs, short YouTube videos, and infographics	Administrative/Executive Representatives from related offices
Coffman, 2009 [50]	None	Qualitative (case study)	State; Legislative	CHBRP faculty and staff, who review and synthesize existing literature	Medical effectiveness and costs of interventions	CHBRP medical effectiveness reports	State legislators
Crowley, 2018 [32]	Research-to-Policy (RPC) Collaboration Model	Mixed Methods; 10 legislative offices and 22 prevention experts were trained	Federal; Legislative	Rapid Response Researcher Network	Research information based on legislative inquiry	In-person meetings and web conferencing	Federal legislative offices; legislators and legislative staff
Friese, 2009 [27]	Two Communities Theory and Community Dissonance Theory	Qualitative (Interviews); 14 interviews	Federal, State; Executive, Legislative	Researchers who presented at Family Impact Seminars between 1993-2002	Child support, early childhood education, helping poor children succeed, long-term care, moving families out of poverty, parenting, prescription drugs, and welfare reform	Presenting or testifying before Congress, legislatures, and committees; responding to individual questions from policymakers and staff via phone and email; serving on committees, advisory panels, and task forces; and writing briefs, memoranda, and contract research reports	Legislators, legislative aides, governor's office staff, legislative service agency staff, and agency representatives
Garcia, 2016 [49]	Consolidated Framework (Damschroder)	Quantitative; 96	County; Executive	Research Evidence (undefined)	Child welfare practices, mental health, juvenile justice, and other social service areas	Academic journals, training manuals, presentations, consultants, intervention developers, and web-based clearinghouse	County-level executive policymakers
Hopkins, 2018 [39]	Social Network Theory	Quantitative; 56 CSS members	State; Executive	CSSS leaders and members	Research related to science education policy	Person-to-person exchanges	State education agency leaders
Jabbar, 2015 [53]	Advocacy Coalition Framework and Operationalization Framework	Qualitative; 53 interviews	State, Local/Municipal; Executive, Legislative	Intermediary organizations, researchers, journalists	Incentive-based reforms for public school systems	Conversations, news stories, press releases, targeted reports, informational cards, blog posts, Twitter	Local, state, and federal policymakers
Jamieson, 1999 [51]	None	Qualitative (case study)	State; Executive	Analysis of Washington state child welfare data	Foster care, guardianship, and racial differences in foster care system	Publications; presentations to a wide array of audiences; repetition of focused messages via presentations, newsletters, news media, public forums, articles, participation in work groups, meetings with key individuals, and broad distribution of reports; placing the data in a local context	DSHS administration, social workers, legislators, judges, the attorney general, tribal councils and American Indian communities, African American communities, child advocates, court-appointed special advocates, and private agencies
Lane, 2011 [33]	knowledge-value mapping (KVM)	Other (Comparative effectiveness study design); (spokespeople for) 6 national organizations	Federal; Executive	Academic journals; training programs and conference proceedings; relevant websites; white papers and internal reports from other sources; experts; Assistive technology agencies	Research on assistive technology devices and services	Electronic media (email, listserv, websites); conferences, presentations, workshops; trainings/certificate programs; electronic media (email, listserv, websites); conference proceedings, presentations, and workshops; white papers and position papers; small-group meetings with policy makers and staff members in government agencies	Public policy agencies
Massell, 2012 [43]	None	Mixed Methods (Interview, Survey); 49 interviews / 300+ surveys	State; Executive	Research; Program evaluation; Research-based guidance; Data on schools in your state; Advice from colleagues within the SEA and external practitioners	Research on improving low-performing schools (broad & generic)	Advice from own colleagues; school data; published original research, research syntheses or summaries, and meta-analyses; results of program evaluations (least often cited)	State education agency leaders
McBride, 2008 [34]	None	Qualitative (focus groups); 3 groups (17, 20+, 20+)	Federal; Legislative	Researchers	Health services research (broadly)	Brochures, fact sheets, working papers/ reports, policy briefs, PowerPoint presentation slides, press releases, Web sites, and other products (provided by researchers)	National Organizations and congressional staffers
McGinty, 2019 [35]	Advocacy Coalition Framework	Qualitative (Interviews, Case Study); 25	Federal, State	Researchers and stakeholders who worked as collaborators in each of the cases	Relevant research for gun policy, opioid policy, and drug control policy	Coalition convening, working groups, sign-on process to endorse recommendations; public release of recommendations; policy dissemination and education via state forums; legislation development; formal policy advocacy; policy implementation support	Policymakers (general)
McVay, 2016 [31]	None	Quantitative; 266	State, Local/Municipal; Executive	Public health researchers (General)	Generalized researched findings (no specific topic)	Face-to-face meetings, academic journals, press releases, policy briefs, and media interviews	Local, state, and/or federal public health departments
Meisel, 2019 [30]	None	Qualitative (interviews); 18 interviews	Federal, State, Other; Other (never specified)	Economic research	Treatment and economic research in substance use disorder	Informal networks with researchers; person-to-person communication; conferences and webinars; literature reviews and summary reports	Policymakers (general)
Mosley, 2012 [52]	None	Qualitative (Interviews, Observations) ; 38 interviews	State; Executive, Legislative	Co-sponsors of bill (9 organizations)	Cost effectiveness & experiences by youth who age out of foster care	Press releases, press conferences, one-page summaries, and other communications; testimonial evidence	State policymakers
Nelson, 2009 [54]	Weiss typology	Qualitative (Interviews, Focus Groups); 10 interviews; 55 participants in 5 focus groups	Federal, State, Local/Municipal; Executive, Legislative	Evidence (peer-reviewed and commissioned studies); Organizations and individuals; Publications and conferences; electronic sources	Educational information influential to No Child Left Behind	Newspapers; media reports; constituent feedback; data (state and local databases, evaluation data from previous initiatives, data collected from multiple databases); personal experience and the experience of others from similar schools, districts, and states; and empirical research evidence	Education policymakers, staff, and advocates
Purtle, 2019 [55]	Weiss typology	Qualitative (Interviews, content analysis of newspaper articles); 44 interviews, unknown # of articles	County; Executive	Published data (County Health Rankings) from the University of Wisconsin Population Health Institute	County-level health information	County Health Rankings Report	County Health Department officials
Sorian, 2002 [58]	None	Qualitative, Quantitative; 292 surveys	State; Legislative, Executive	Publications and conferences; electronic sources; organizations (public and private) and individuals	General health policy issues	Electronic and hard copy print materials	State legislative policymakers
Valentine, 2014 [41]	None	Qualitative (Interviews, Focus Groups)	State; Executive	Senior advisors from to policymakers from state departments of mental health in management positions and Policy director from a national nonprofit organization	State-level mental health disparities	Mental health care disparity report cards	State mental health executive; audience for mental health care disparity report cards would be state policymakers
Weiss, 2008 [29]	Weiss typology "Imposed Use"	Qualitative (Interviews); 16 interviews	Federal, State, Local/Municipal; Executive	US Department of Education expert panel	Recommendations for substance abuse prevention programs for implementation in primary and secondary educational settings	List of Exemplary and Promising Prevention Programs	School district stakeholders
Weissman, 2015 [42]	None	Quantitative; 46 states, 60 (out of 100) directors	State; Executive	Published research; experts; own data; patients	Application of CER coverage decisions	RCTs; Consensus statements; Systematic Reviews; Expert Opinion; Observational studies (own data; other data); patient experience/consumer advocacy	State Medicaid Directors and Pharmacy Directors
Yanovitsky, 2019 [36]	Message Framing Theory; Weiss typology	Qualitative; Thematic and content analysis of 786 documents	Federal; Legislative	Government, academic, think-tank research, anecdotal evidence, and 'generic'	Research evidence on childhood obesity	Congressional committee hearings & bills	Federal legislators and their staff
Zelizer, 2018 [44]	None	Quantitative; 74 legislators (1,216 legislator-bill dyads)	State; Legislative	Legislative staffer who worked for the Veterans Caucus	Information on proposed legislation	In-person meeting with one Veterans caucus staffer	State senator or representative

We examined dissemination based on geographic regions and/or political boundaries (i.e., regions or states). Sixteen of the 27 studies (about 59%) used national samples or multiple states and did not provide geographic-specific results [27–42]. Two studies (about 7%) did not specific the geographic region or state in which the study took place [43, 44]. Of the remaining studies, four examined policymaking in the Northeastern United States [45–48], four in the Western US [49–52], and one in the South [53]. The geographic regional groups used similar channels to disseminate evidence to policymakers including publications and presentations.

We also analyzed whether dissemination at different levels of government (i.e., local, state, and federal) used unique channels. Six of included studies (about 22%) examined multiple levels of government and did not separate results based on specific levels of government [27–31, 53]. One study did not specifically identify the level of government used [46]. While there is considerable overlap in dissemination channels used at each level of government, there are some unique characteristics.

Five studies (about 18.5%) examined dissemination at the federal level [32–36]. At the federal level, dissemination channels tended to be more formal such as congressional committee hearings [36] and legislative development [35]. Twelve studies (about 44%) evaluated dissemination at the state level [38–44, 47, 48, 50–52]. State level dissemination heavily relied on printed materials including from mental health care disparity report cards [41], policy briefs [38], and effectiveness reports [50]. Another common channel was in-person communications such as one-on-one meetings [44] and presentations to stakeholders [51]. Three studies (about 11%) focused on local-level government. Dissemination channels at the local level had little consistency across the three studies with channels including public education [45], reports [37], and print materials [49].

Roughly half of studies were atheoretical (*n* = 13). Four studies used the Weiss Typology [29, 36, 54, 55], two studies used the operationalization framework [45, 53], and two studies used the advocacy coalition framework [53, 56].

### Model for dissemination of research

**Fig. 3:**
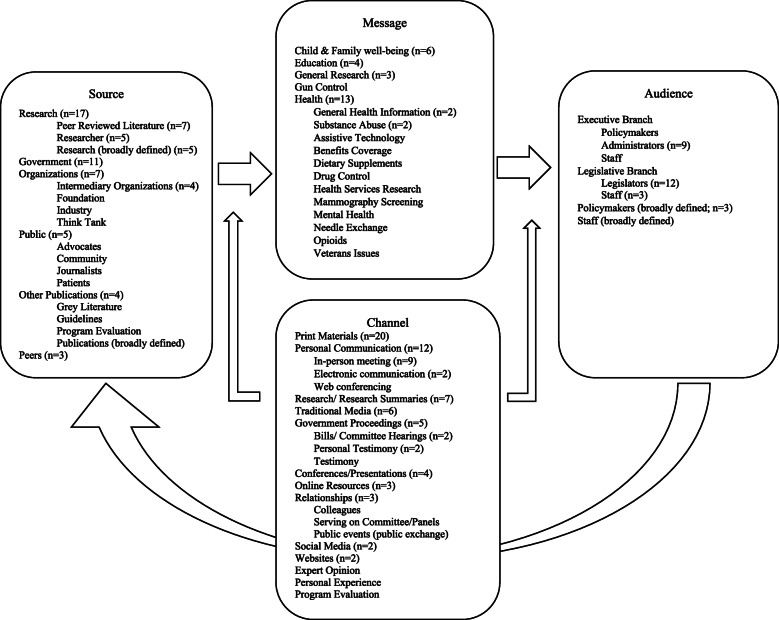
A conceptual model for dissemination of research to policymakers. The populated conceptual model builds on the Model for Dissemination of Research by organizing findings from the current systematic review to build an understanding of how research is disseminated to policymakers in the United States

We used the Model for Dissemination of Research to summarize the findings from the included studies into the themes of source, message, audience, and channel (i.e., strategies). We integrated themes from the studies into the Model (see Fig. 3).

#### Source

The sources of knowledge varied across studies with some studies including multiple sources of social policy information. The most common sources of knowledge included research, as in peer-reviewed literature (*n* = 7) [30, 33, 38, 42, 43, 49, 54], researchers (*n* = 5) [27, 31, 32, 34, 56], and research broadly defined (*n* = 5) [36, 39, 47, 48, 55], the government (*n* = 11) [29, 36, 41–44, 47, 50, 54, 56, 57], and organizations (*n* = 7) [33, 36, 46, 52–54, 56].

#### Message

The majority of studies focused on health topics (*n* = 12) [29, 30, 33, 34, 38, 41, 42, 45, 47, 55, 56, 58] and child and family well-being (*n* = 6) [27, 36, 46, 49, 52, 57]. The remaining studies covered the topics of education (*n* = 4) [39, 43, 53, 54], guns [56], veterans [44], and general social research (*n* = 3) [31, 32, 48]. Multiple studies offered specific recommendations for message framing, suggesting that the packaging of information is as critical as the information itself [27]. One study piloted multiple styles of policy briefs and found staffers preferred to use and share narrative or story-based briefs while legislators were more likely to use and share statistical, data-based briefs [38]. This finding was mirrored in two studies that found testimonial or descriptive evidence to be as effective as data-driven research [34, 52], particularly in the context of sympathetic populations [52]. Three studies highlighted the reliance of effective message delivery on the message’s ability to capture audience interest (e.g., what the research means to the policymaker, specifically and if possible, personally) [27, 34, 41]. Finally, two studies emphasized creating a sense of urgency or even shock-value within the message in order to capture policymakers’ interest [36, 57].

#### Audience

The audience included executive branch policymakers [49], administrators (*n* = 9) [27, 31, 38, 39, 41, 43, 53, 55, 57], and staff [42]. Studies which focused on the legislative branch examined legislators (*n* = 12) [27, 32, 36, 38, 44–47, 50, 52, 53, 58] and staff (*n* = 3) [32, 34, 36]. Three studies examined broadly defined policymakers [33, 54, 56] and generalized staff [54] without indication for specific branch of government.

#### Channel

Included studies examined a variety of channels with many including multiple channels. Print materials was the most commonly used channel, including reports (*n* = 10) [27, 30, 33, 41, 46, 50, 53, 55, 57, 58] and policy briefs (*n* = 3) [31, 34, 38]. Researchers examined in-person meetings and communications as a channel to disseminate research (*n* = 9) [30, 32, 33, 39, 44, 48, 53, 56, 57]. Research and research summaries were also studied (*n* = 7) [30, 31, 42, 47, 49, 52, 54]. Both traditional (*n* = 6) [31, 33, 47, 52–54] and social media (*n* = 2) [47, 53] were examined as channels to disseminate research to policymakers. Other channels include conferences and presentations (*n* = 4) [33, 34, 49, 57], electronic communication (*n* = 2) [27, 57], online resources (*n* = 3) [34, 49, 58], and personal testimony (*n* =2) [42, 52].

### Effectiveness and lessons learned

The majority of studies employed qualitative research methods (e.g., interviews, case studies, focus groups) to evaluate the impact of scientific research on domestic social policy. Our review of the literature also identified nine quantitative and mixed-methods studies [31, 32, 38, 39, 42–44, 49, 58]. We identified a series of cross-cutting dissemination strategies for engaging policymakers including recommendations for and barriers to research-to-policy (see Table 2).

**Table 2:** Effectiveness and lessons learned

Strategy for engaging policymakers	Recommendations for research-policy translation	Barriers to research-policy translation
Start early	• Engage policymakers when planning research [34, 41] • Be strategic about research and audience [29, 40] • Take initiative to contact policymakers [44]	
Drum up support	• Involve a broad pool of experts [35] • Cultivate broad coalition of supporters [47]	• Policymakers may appear not to value research [28]
Use research evidence 'champions' or 'brokers'	• Research use 'champions' engage with community stakeholders and policymakers [45] • Intermediary organizations connect “research supply” to “research demand” [53] • External brokers play a role both in connecting policymakers to research and in conceptualizing and developing policy [39, 43]	• Intermediary individuals or organizations may select or spin research to make their point [45, 52, 53] • Policymakers may have a list of preferred evidence brokers [53] • Basing policy on evidence requires identified 'best evidence', which may reflect bias and favoritism [29]
Context matters	• Integrate research evidence into broader sociopolitical context [45] • Research must be locally, contextually relevant [54, 55, 57] • Specify which government office(s) are responsible [47]	• Federally imposed policies (e.g., education) often override local expertise around context and population [29] • Ideology, whether personal or regional, may create a barrier between researchers and policymakers [27, 41, 44, 50, 54–56]
Make research products timely, relevant, and accessible	• Tailor design of products to meet diverse end user needs [27, 34] • Present research in commonly-used formats (e.g., briefs, talking points, videos) [48] • Research must be timely and geared to policymakers' concerns [27, 38, 42, 44, 52] • Use clear, careful language [27] • Formalize the organizational / individual process of translating research to policy [32]	• Complexity of research [56] • Disconnect between the goals and language of policymakers and researchers [41, 52, 56] • Concerns about data/research evidence quality [29, 41, 42]
Know the players and the process	• Familiarize yourself with policymaking process [27, 31] • Show respect for policymakers' knowledge/experiences [27] • Learn about / build relationships with the target policymaking audience [27, 30] • Expand contact and working relationships with end users [34]	• Lack of familiarity with effective dissemination strategies [31] • Lack of financial and institutional support for dissemination [31]
Miscellaneous	• Approach policy work as an educator rather than as an advocate [27]	

#### Start early

Four studies highlighted the importance for early and ongoing engagement with policymakers throughout the research process in order to maximize interest and applicability. Researchers are encouraged to take the initiative to contact policymakers as early as possible in the research process. Many policymakers may be interested in accessing and using research but uncertain who or how to make connections in the academic or research community [27]. Involving policymakers when designing projects and framing initial research questions increases the likelihood that key policy stakeholders will remain invested in the work by allowing their individual research interests to shine [34, 41]. Early engagement also ensures that research products (e.g., reports, policy briefs, factsheets) will have strategic usefulness for policymakers [30].

#### Drum up support

In addition to early policymaker engagement, three studies highlighted the need for researchers to garner outside support for their work, ideally involving a broad pool of experts and cultivating a broader coalition of supporters than typical academic endeavors [47]. Often, policymakers appear unwilling or uninterested in considering the application of evidence to their work [45, 53]; when researchers can demonstrate the value and relevance of their work [58], policymakers may be more likely to engage.

#### Use research evidence “champions” or “brokers”

A common strategy for garnering support (as recommended above) is the use of evidence *champions* or *brokers*; these are intermediary individuals or organizations who connect research suppliers (e.g., individual researchers, academic institutions) to research demand (e.g., policymakers) [53]. These champions can broker important connections; however, researchers and policymakers alike must remember that these intermediaries are not neutral carriers of information, and may spin research in support of personal agendas [45, 52, 53]. Individual biases may also present a barrier in research-to-policy translation, as individuals or organizations are empowered to select the “best” research evidence to share with policymakers [29]. One study found that nearly half of state policymakers named professional associations as trusted sources for research information, specifically because the organization is perceived not to have a stake in the final policy outcome [58].

Two studies specifically addressed the role of intermediary organizations or brokers in the translation of research evidence to policy. Hopkins et al. [39] explored the exchange of research evidence among state education agency (SEA) leaders, while Massell et al. [43] examined more broadly the origins of research evidence use in three SEAs. Both studies found that external brokers played a role in connecting SEA policymakers to relevant research, as well as in the conceptualization and development of policy.

#### Focus on context

Multiple studies stressed the importance of research evidence being contextually relevant to the specific policy audience [29, 54, 55, 57]. For some policymakers, the needs and interests of local constituents will drive the use of research and the specifics of the policy agenda; for others, discussions that integrate research evidence into the broader sociopolitical context will be more effective [45]. For state- and local-level policymakers, policies may be most effective when based on the evidence-based understanding of local stakeholders, rather than imposed from the federal level without local contextual details [29].

Ideology of external advisors and brokers (as discussed above) and policymakers’ own personal beliefs and experiences [54] and the prevailing political ideology of a particular geographic region [55] are critical components of context. Ideological beliefs, often deeply held and personal, may create a barrier between researchers and policymakers [41], though differentiating ideology from other factors that affect individual position-taking is difficult in most situations [44]. McGinty et al. [56] suggest that in polarized contexts involving strong ideological beliefs, research may add legitimacy to a particular viewpoint, though as with brokers, that research is likely to be carefully curated to support the desired message. Purtle et al. [55] concur, reporting that some county health officials were wary of the potential to spin research findings to make a case for certain programs over others and noted the need to avoid the challenge of distorting evidence. Two studies recommend positional neutrality as a researcher’s best approach to handling potential ideological differences, suggesting that presenting research findings as simple fact, rather than making specific recommendations for action, may help avoid conflict and also help researchers gain credibility across the ideological spectrum [27, 50].

#### Make research products timely, relevant, and accessible

As with all research endeavors, timeliness and relevance are paramount. However, the typical timeline for academic research (years) is often too long for policymakers whose window for championing a policy action is much shorter (weeks or months) [27, 52]. A frequently reported barrier in research-to-policy translation is the complexity of research and concerns about the quality of research evidence [29, 41, 56]; one strategy for combating this concern is the use of clear, careful language [27], and tailored, audience-specific products that meet the needs of a diverse population of end users [27, 34, 58]. Research that is presented in commonly used, accessible formats (e.g., briefs, factsheets, videos) [48] may also be more effective, though one study found that use of these formats was dependent on job type, with legislators and staffers preferring different formats [58].

Multiple studies engaged with policymakers in an effort to determine how they receive research evidence and what strategies or formats are most desirable or effective [38]. After piloting four different styles of policy briefs (on the same research topic) with state-level policymakers, Brownson et al. [38] found that while all styles of brief were considered understandable and credible, opinions on the usefulness of the brief varied by the style of the brief and by the level of policymaker (e.g., legislative staff, legislators, and executive branch administrators). These findings suggest that targeted, audience-specific research evidence materials may be more likely to be used by policymakers than generic research evidence. One study explored the usefulness of electronic vs. printed research material and again found differences by type of policymaker—legislators were more likely to read hard copy printed material, while staffers gave higher ratings to online content. Not surprisingly, the age of the policymaker also played a role in the choice to access electronic or printed material, with younger policymakers much more likely to read electronic copy than were their older peers [58].

A study on state policymakers’ perceptions of comparative effectiveness research (CER) found that the most useful research is that which is consistent and specific to the needs of the policymakers [42]. The same study identified related barriers to the use of CER in policy decision-making, citing a lack of relevant high quality or conclusive research [42].

Finally, two studies described pilot projects focused on the delivery of research evidence directly to policymakers. The first cultivated researchers’ capacity to accelerate the translation of research evidence into useable knowledge for policymakers through a rapid response researcher network [32]. This model was shown to be effective for both researchers (in mobilizing) and policymakers (in eliciting requests for research evidence to bolster a policy conversation or debate) [32]. The second implementation study reported on a field experiment in which state legislators randomly received relevant research about pending policy proposals [44]. Findings from this study suggest that having relevant research information increases policymakers’ co-sponsorship of proposals by 60% and highlights the importance of research access in the policy process [44].

#### Know the players and the process

Policymakers are as much experts in their arena as researchers are in their academic fields. In order to build lasting working relationships with a target policymaking audience and maximize the relevance of research products for policy work, researchers must first understand the policy process [27, 30, 34]. One study examined the role of researchers themselves in disseminating findings to policymakers and identified individual- and organizational-level facilitators and barriers to the process [31]. Researchers’ familiarity with the policy process, the relevance of policy dissemination to individual programs of research, and the expectation of dissemination (from higher institutional or funding bodies) facilitated the research-to-policy exchange, while lack of familiarity with effective dissemination strategies and lack of financial and institutional support for dissemination emerged as primary barriers in the research-to-policy exchange [31].

## Discussion

Public policy, whether legislative, executive, or judicial, affects all areas of daily life in both obvious and subtle ways. The policy process (i.e., the steps from an idea to policy enactment) does not exist in a vacuum; it is influenced by many factors, including public opinion [59, 60], special interest groups [61], personal narratives [62], expressed needs of constituents [1], the media [63–65], and corporations [66, 67]. Research may also play a role in shaping policy and has the potential to add objectivity and evidence to these other forces [1, 2, 68]. The current study synthesizes existing knowledge to understand dissemination strategies of social policy research to policymakers in the United States.

Many channels exist to disseminate evidence to policymakers, with the most common being print materials (i.e., reports and policy briefs). This finding is surprising in our current digital age, as print materials are necessarily time-bound and rapidly evolving technology has created more channels (e.g., social media, videos) which may be preferred by policymakers. This shift creates an opportunity to optimize the content of print materials to disseminate in new mediums; it also offers a chance for authors to improve the accessibility of their work for broader audiences (e.g., via more visual presentation formats) [15, 69–71].

Our review found strategies to increase effectiveness of research dissemination to policymakers includes starting early, drumming-up support, using champions and brokers, understanding the context, ensuring timeliness, relevance, and accessibility of research products, and knowing the players and the process. These themes align with existing knowledge about policymaker preferences including face-to-face engagement [72, 73], contextual considerations (e.g., timeliness and budget) [2, 72], and existing barriers and facilitators to research evidence use [4, 5]. Our study adds to what we already know about policymakers’ desire for research evidence and their varying preferences as to the context and form of that knowledge [2, 72, 74] and supports existing efforts to bridge the gap between researchers and policymakers.

Many of the barriers and facilitators to research dissemination that we identified in this review mirror those cited by policymakers as barriers and facilitators to evidence use; this overlap reasonably suggests that efforts to expand research dissemination may improve the other. Particularly relevant lessons from the evidence use literature that also emerged from our review include emphasis on the benefit of building personal relationships between researchers and policymakers [5, 75, 76], narrowing the perceived gap between the two groups [77, 78], and changing the culture of decision making to increase appreciation for the value of research in policy development [5, 75–77]. Considering the multiple pathways through which research evidence is used in policy, from providing direct evidence of a program’s effectiveness to informing or orienting policy makers about relevant issues [23], these shared lessons around barriers and facilitators may better inform researchers, policymakers, and staff as to best practices for future communication and collaboration.

Our findings also highlight several unique elements of the US policy landscape, wherein significant power is reserved from the federal-level and afforded to state-level government. In some states, this power is further distributed to county and local governments. This system creates major variation across the country in both policy decisions and in resource availability for social policy implementation. Despite our relatively unique government structure, however, many of the effective strategies for dissemination we identified mirror strategies found in other countries [79, 80].

Studies that focused on a specific level of government had some unique characteristics such as formality and reliance on print materials. For example, federal dissemination relied more heavily on formal legislative testimony while state level material relied on written policy materials (e.g., policy briefs, report cards). However, these results are limited by small sample sizes and limited evidence about effectiveness.

A wide range of contextual variables may influence policy dissemination in the US at different levels of government. In the federal legislative context alone, multiple committees and subcommittees of both the U.S. House of Representatives and the U.S. Senate may exercise some control over programs and policies related to a single social policy issue (e.g., child and family services) [81]. At the federal level, the Congressional Research Service (CRS) provides non-partisan research support to legislators in multiple formats including reports on major policy issues, expert testimony, and responses to individual inquiries; the Domestic Social Policy Division offers Congress interdisciplinary research and analysis on social policy issues [82]. While there may be fewer decision-makers for each issue on the state level, policymaking is further complicated by the extensive rules and reporting requirements attached to state use of federal funding as well as competing priorities or needs at the local level within each state [83, 84]. Another dissemination influence may include geographic proximity; for example, geographical proximity may increase the likelihood of university-industry partnerships [85].

Infrastructure differences may also represent important differences between the US social policy context and that of other developed nations. Each country has a distinct and perhaps unique policy context given available resources, political rules and regulations, and priorities. While models for infrastructure and dissemination interventions may be shared across policy contexts, it may be difficult to directly compare dissemination strategies in one country with dissemination strategies in another country.

Several examples across western countries contribute to a stronger nexus between research evidence and the policy-making process. In the United States, the Wisconsin Family Impact Seminars (www.wisfamilyimpact.org) are an example of long-standing initiatives that provide the opportunity for researchers and policymakers to come together to discuss unbiased policy-relevant evidence [86]. As exemplified by Friese and Bogenschneider [27], these forums continue to be perceived as objective, relevant, and useful by policymakers and have succeeded at bringing attention to social policy [86]. Researchers and policymakers in Canada have sought to bridge the research-to-policy gap. For example, the Canadian Foundation for Healthcare Improvement (formerly the Canadian Health Services Research Foundation), funded by the Canadian federal government, brings together researchers and policymakers early and throughout the research development process to discuss, prioritize, and evaluate opportunities for research and dissemination [79]. In the UK, infrastructure at the national level includes the National Institute for Health Research Policy Research Programme, which funds health research with the explicit goal of informing national policy decisions in health and social care [87]. These efforts include open calls for research proposals as well as 15 dedicated Policy Research Units located at leading academic institutions around the country. Another resource is the EPPI-Centre at University College London, which provides policymakers support for finding and using research to inform policy decisions through its Research Advisory Service. This allows researchers to work alongside policymakers to reach their goals in addressing educational needs with evidence-informed policy [80].

### Limitations

The current study has several limitations—these illustrate opportunities for future research. First, we attempted to cast a wide net when searching for studies which examined the influence of research on social policy by including a broad search of the peer-reviewed literature, think tanks, and content experts. However, it is possible we missed some studies which examine how research influences policy. Second, we provide a rationale for focusing on US studies and that our findings may not be generalizable to other countries. Third, we were unable to assess the risk of bias for individual studies as current standards note difficulties in assessing quality and bias in qualitative research [88]. Fourth, many studies examined multiple channels or strategies for how research influences policy, so the parsing of singular strategies (e.g., policy brief, in-person meeting) as an effective approach should be interpreted with caution. Additional investigation is needed to explore and test causal pathways in *how* these channels can best influence social policy. Fifth, the majority of studies did not use any theory or framework as a foundation or guide for exploration. This gap may indicate a space to use frameworks such as the Model for Dissemination of Research to guide future research. Finally, the dearth of mixed-methods studies that systematically evaluate the impact of research evidence on domestic social policy (this review identified only 3) presents an opportunity for future work in this field to integrate quantitative and qualitative methodologies.

One significant challenge to increasing the rigor in dissemination research studies is the difficulty in choosing and then measuring an outcome. Many of the studies included in this review are either case studies or descriptive, making it difficult to determine what, if any, impact the given research had on policy. Bogenschneider and Corbett discuss this at length as one of the primary challenges to furthering this research [72], imploring researchers not to focus solely on the outcome of whether or not a piece or legislation passes but rather to examine whether research influenced one of the proposed policy options [72]. However, this information can be difficult both to operationalize and to collect. That said, some researchers have already begun to think beyond the passage of legislation, as evidenced by Zelizer [44] who examined bill co-sponsorship rather than passage. A recent review of health policy implementation measurement found that validated quantitative measures are underutilized and recommends further development and testing of such measures [89]. Difficulties in identifying robust outcomes and high-quality scales to operationalize them present opportunities for additional exploration in this area.

Dissemination and implementation are often described together; not surprisingly, is overlap in effective strategies for each. The current review identified six dissemination strategies and described their reported effectiveness, while the Expert Recommendations for Implementing Change (ERIC) Project identified 73 implementation strategies [90]. One such similarity is obvious: the dissemination strategy of using champions and brokers mirrors the ERIC implementation strategy of identifying and preparing champions. The difference between the number of implementation strategies and dissemination strategies is striking and highlights the gap in research. Future work should further explore the degree to which dissemination strategies and implementation strategies either overlap or are distinct.

Finally, the dissemination of research to policymakers may raise certain ethical issues. It is imperative for researchers to critically assess when and how to disseminate research findings to policymakers, keeping in mind that promoting a specific policy agenda may result in a perceived or real loss of objectivity [91]. Syntheses of policy-relevant evidence can be useful, particularly when researchers work in partnership with non-governmental organizations to inform the policy process.

## Conclusions

We summarize strategies and illuminate potential barriers to the research-to-policy dissemination process. Key findings are drawn from multiple disciplines and suggest that lessons learned may cut across both research topics and levels of government. The most frequently referenced channel for dissemination to policymakers was print materials, with personal communication (including both in-person and electronic meetings and individual communications) a close second. Corresponding strategies for effective dissemination to policymakers included starting early, drumming-up support, using champions and brokers, understanding the context, ensuring timeliness, relevance, and accessibility of research products, and knowing the players and the process. A shared feature of these strategies is the distillation of complex research findings into accessible pieces of relevant information that can then be delivered via multiple avenues.

Interdisciplinary collaboration is a common practice in scientific research [92]. Our findings provide leads on how to more effectively to engage with policymakers, leading to a greater likelihood of translating research evidence into policy action. Engaging policymakers early as contributing members of the research team, maintaining communication during the research process, and presenting relevant findings in a clear, concise manner may empower both researchers and policymakers to further apply scientific evidence to improve social policy in the United States.

## Supplementary information

**Additional File 1.** PRISMA Checklist.

**Additional File 2.** Search Strategy.

**Additional File 3.** Data Abstraction Form.

## Data Availability

Raw search results, citations, and abstracts available upon request.

## Abbreviations

US: United States
PRISMA-P: Preferred Reporting Items for Systematic Reviews and Meta-Analyses
CER: Comparative Effectiveness Research
ERIC: Expert Recommendations for Implementing Change
