# Supplementary material for: Strategies for effective dissemination of research to United States policymakers: a systematic review
Source: Implement Sci. 2020 Oct 15;15:89. doi: 10.1186/s13012-020-01046-3 (PMC7560305; doi:10.1186/s13012-020-01046-3)
Supplement: Supplementary file 2 — Additional File 2. Search Strategy. [file 13012_2020_1046_MOESM2_ESM.docx]

**Additional File 1: Search Strategy**

Database: Web of Science

1980 to 2019

1. ((TS=(research OR study OR studies OR knowledge)) AND LANGUAGE: (English)

*Indexes=SCI-EXPANDED, SSCI, A&CHI, ESCI TIMESPAN=1980-2019*

1. ((TS=(policy OR policies OR law OR laws OR legislation)) AND LANGUAGE: (English)

*Indexes=SCI-EXPANDED, SSCI, A&CHI, ESCI TIMESPAN=1980-2019*

1. ((TS=(use OR utilization OR utilisation)) AND LANGUAGE: (English)

*Indexes=SCI-EXPANDED, SSCI, A&CHI, ESCI TIMESPAN=1980-2019*

1. ((TI=(disseminate OR dissemination OR disseminating)) AND LANGUAGE: (English)

*Indexes=SCI-EXPANDED, SSCI, A&CHI, ESCI TIMESPAN=1980-2019*

1. ((TI=(implementation OR implementing OR implement)) AND LANGUAGE: (English)

*Indexes=SCI-EXPANDED, SSCI, A&CHI, ESCI TIMESPAN=1980-2019*

1. ((TI=(translate OR translation OR translating)) AND LANGUAGE: (English)

*Indexes=SCI-EXPANDED, SSCI, A&CHI, ESCI TIMESPAN=1980-2019*

1. #3 AND #2 AND #1
2. #6 OR #5 OR #4
3. #8 AND #7
4. (#8 AND #7 AND CU=USA) AND LANGUAGE: English)

*Indexes=SCI-EXPANDED, SSCI, A&CHI, ESCI TIMESPAN=1980-2019*

Database: Academic Search Premier

1980 to 2019

English

United States

Boolean/Phrase

AB ( AB ( research OR study OR studies OR knowledge OR evidence ) AND ( policy OR policies OR law OR laws OR legislation ) AND ( use OR utilization OR utilisation ) OR ( disseminate OR dissemination OR disseminating ) OR ( implementation OR implementing OR implement ) OR ( translate OR translation OR translating ) ) AND KW ( AB ( research OR study OR studies OR knowledge OR evidence ) AND ( policy OR policies OR law OR laws OR legislation ) AND ( use OR utilization OR utilisation ) OR ( disseminate OR dissemination OR disseminating ) OR ( implementation OR implementing OR implement ) OR ( translate OR translation OR translating ) )

KW ( ( research OR study OR studies OR knowledge OR evidence ) AND ( policy OR policies OR law OR laws OR legislation ) AND ( use OR utilization OR utilisation ) OR ( disseminate OR dissemination OR disseminating ) OR ( implementation OR implementing OR implement ) OR ( translate OR translation OR translating ) ) AND AB ( ( research OR study OR studies OR knowledge OR evidence ) AND ( policy OR policies OR law OR laws OR legislation ) AND ( use OR utilization OR utilisation ) OR ( disseminate OR dissemination OR disseminating ) OR ( implementation OR implementing OR implement ) OR ( translate OR translation OR translating ) ) )
