# Supplementary material for: Strategies for effective dissemination of research to United States policymakers: a systematic review
Source: Implement Sci. 2020 Oct 15;15:89. doi: 10.1186/s13012-020-01046-3 (PMC7560305; doi:10.1186/s13012-020-01046-3)
Supplement: Supplementary file 3 — Additional File 3. Data Abstraction Form. [file 13012_2020_1046_MOESM3_ESM.docx]

**Additional File 2: Data Abstraction Form**

1. Name of Reviewer
2. General Article Information
   1. Title
   2. First Author (Last Name only)
   3. Year of Publication
3. What theory or framework guides this research? (Name OR simple explanation if no name)
4. Research Question(s) (Copy and paste with page number)
5. Methods
   1. Qualitative
   2. Quantitative
   3. Mixed Methods
   4. Other
6. Research Strategy
   1. Interview
   2. Survey
   3. Case Study
   4. Focus Groups
   5. Other
7. Sample Size
8. Unit of Analysis
9. Level of Government
   1. Federal
   2. State
   3. County
   4. Local/Municipal
   5. Other
10. Branch of Government
11. Geographic Location
12. Data Type
    1. Primary Data Collection
    2. Secondary Data Collection
13. Dependent Variable(s)
14. Independent Variable(s)
15. Source (i.e. where/who is the information coming from)
16. Message (i.e. what information is being shared)
17. Channel (i.e. how is the information getting to the target audience; e.g. policy brief, in person conversation)
18. Audience (i.e. who is the information going to)
19. Measures/Descriptions of effectiveness and results.
20. Any terminology used that may be helpful (copy and paste with page number)
